# Supplementary material for: Optimized Vivid-derived Magnets photodimerizers for subcellular optogenetics in mammalian cells
Source: eLife. 2020 Nov 11;9:e63230. doi: 10.7554/eLife.63230 (PMC7735757; doi:10.7554/eLife.63230)
Supplement: Supplementary file 1. — The organelle‐targeting sequences (OTS) used and their position, the fluorescent tag, and the original or mutant Magnets used in each construct are indicated. [file elife-63230-supp1.docx]

**Supplementary File 1: Constructs used to express wild-type or mutant Magnets on different subcellular compartments.**

The Organelle‐Targeting Sequences (OTS) used and their position, the fluorescent tag, and the original or mutant Magnets used in each construct are indicated.

| **Name** | **Localization** | **OTS position** | **Origin** | **NCBI Accession Number** | **Fused amino acid sequence** | **Fluorescent tag** |
| --- | --- | --- | --- | --- | --- | --- |
| **nMagHigh1-EGFP-Mito** | Mitochondria | C-ter | Outer membrane protein OMP25 or synaptojanin-2-binding protein (Homo sapiens) | NP_060843.2 | VQNGPIGHRGEGDPSGIPIFMVLVPVFALTMVAAWAFMRYRQQL | EGFP |
| **eMagA^F^-EGFP-Mito** | Mitochondria | C-ter | Outer membrane protein OMP25 or synaptojanin-2-binding protein (Homo sapiens) | NP_060843.2 | VQNGPIGHRGEGDPSGIPIFMVLVPVFALTMVAAWAFMRYRQQL | EGFP |
| **eMagA-EGFP-Mito** | Mitochondria | C-ter | Outer membrane protein OMP25 or synaptojanin-2-binding protein (Homo sapiens) | NP_060843.2 | VQNGPIGHRGEGDPSGIPIFMVLVPVFALTMVAAWAFMRYRQQL | EGFP |
| **ER -EGFP-eMagA** | Endoplasmic reticulum | N-ter | Cytochrome P450 2C1 (Oryctolagus cuniculus) | AAA31436 | MDPVVVLGLCLSCLLLLSLWKQSYGGG | EGFP |
| **Lys-eMagA-EGFP** | Lysosomes | N-ter | Ragulator complex protein LAMTOR1  (Homo sapiens) | NP_060377.1 | MGCCYSSENEDSDQDREERKLLLDPSSPPTKALNGAEPNY | EGFP |
| **eMagA^F^-EGFP-PM** | Plasma membrane | C-ter | Isoform 2B of GTPase KRas  (Homo sapiens) | P01116-2 | KKKKKKSKTKCVIM | EGFP |
| **ER-mCherry-eMagA** | Endoplasmic reticulum | N-ter | Cytochrome P450 2C1 (Oryctolagus cuniculus) | AAA31436 | MDPVVVLGLCLSCLLLLSLWKQSYGGG | mCherry |
| **Lys-eMagB-iRFP** | Lysosomes | N-ter | Ragulator complex protein LAMTOR1  (Homo sapiens) | NP_060377.1 | MGCCYSSENEDSDQDREERKLLLDPSSPPTKALNGAEPNY | iRFP670 |
| **eMagB-iRFP-Mito** | Mitochondria | C-ter | Outer membrane protein OMP25 or synaptojanin-2-binding protein (Homo sapiens) | NP_060843.2 | VQNGPIGHRGEGDPSGIPIFMVLVPVFALTMVAAWAFMRYRQQL | iRFP670 |
| **eMagA-mCherry-Mito** | Mitochondria | C-ter | Outer membrane protein OMP25 or synaptojanin-2-binding protein (Homo sapiens) | NP_060843.2 | VQNGPIGHRGEGDPSGIPIFMVLVPVFALTMVAAWAFMRYRQQL | mCherry |
