# Supplementary material for: Optimized Vivid-derived Magnets photodimerizers for subcellular optogenetics in mammalian cells
Source: eLife. 2020 Nov 11;9:e63230. doi: 10.7554/eLife.63230 (PMC7735757; doi:10.7554/eLife.63230)
Supplement: Supplementary file 2. [file elife-63230-supp2.docx]

**Supplementary File 2: Constructs encoding the soluble prey proteins used in this study.**

| **Name** | **Intracellular Localization** | **Functional Domain and Position** | **Origin** | **NCBI Accession Number** | **Fluorescent tag** |
| --- | --- | --- | --- | --- | --- |
| **pMagFast2-TagRFP-T** | Cytosolic | N/A |  |  | TagRFP-T |
| **eMagB^F^ -TagRFP-T** | Cytosolic | N/A |  |  | TagRFP-T |
| **eMagB-TagRFP-T** | Cytosolic | N/A |  |  | TagRFP-T |
| **mCherry- eMagB^F^ -5ptase_OCRL_** | Cytosolic | C-term | [OCRL inositol polyphosphate-5-phosphatase (OCRL), Homo sapiens](https://blast.ncbi.nlm.nih.gov/Blast.cgi#alnHdr_1677498650) | [NM_001587.4](https://www.ncbi.nlm.nih.gov/nucleotide/NM_001587.4?report=genbank&log$=nucltop&blast_rank=1&RID=1ZNZKK5V014) | mCherry |
| **TagRFP-T-VAPB(1-218)- eMagB** | Cytosolic | Internal | VAMP associated protein B (VAPB 1-218), Homo sapiens | [NM_004738.5](https://www.ncbi.nlm.nih.gov/nucleotide/NM_004738.5?report=genbank&log$=nuclalign&blast_rank=9&RID=9YPMA0BE016) | TagRFP-T |
| **TagRFP-T-eMagB-PH_OSBP87-126_** | Cytosolic | C-term | Homo sapiens oxysterol binding protein (OSBP), mRNA | NM_002556.3 | TagRFP-T |
