# Supplementary material for: Optimized Vivid-derived Magnets photodimerizers for subcellular optogenetics in mammalian cells
Source: eLife. 2020 Nov 11;9:e63230. doi: 10.7554/eLife.63230 (PMC7735757; doi:10.7554/eLife.63230)
Supplement: Supplementary file 3. [file elife-63230-supp3.docx]

**Supplementary File 3: Mutants tested**

| **Amino acid substitutions introduced in both**  **pMagFast2 and nMagHigh^M55A^** | **Heterodimerization observed after preincubation of cells at various temperatures** | | | | **NOTES** |
| --- | --- | --- | --- | --- | --- |
|  | **28°C** | **33°C** | **35°C** | **37°C** |  |
| **WT** | **YES** | **NO** | **NO** | **NO** |  |
| **G49A/Y50F** | **NO** | **NT** | **NT** | **NO** |  |
| **G49A/Y50I** | **NO** | **NT** | **NT** | **NO** |  |
| **Y94E/N100E** | **YES (-)** | **NO** | **NO** | **NO** |  |
| **N130K/N133K** | **YES (+)** | **YES** | **NO** | **NO** |  |
| **L64I/V67I/T69L** | **YES (+)** | **YES** | **NO** | **NO** | **Slower off kinetics (t_1/2_= 5.5 mins)** |
| **L64I/V67I/T69M** | **NO** | **NT** | **NT** | **NO** |  |
| **S99N** | **YES** | **YES** | **NO** | **NO** |  |
| **S99N/R106K** | **NO** | **NO** | **NT** | **NO** |  |
| **T123R/K125R/D128R/N130D** | **NO** | **NT** | **NT** | **NO** |  |
| **N56T** | **NO** | **NO** | **NO** | **NO** |  |
| **L64I** | **YES (-)** | **NO** | **NO** | **NO** |  |
| **V67I** | **YES (+)** | **NO** | **NO** | **NO** | **Slower off kinetics** |
| **T69L** | **YES (+)** | **NO** | **NO** | **NO** |  |
| **F162I** | **NO** | **NO** | **NO** | **NO** |  |
| **F162L** | **NO** | **NO** | **NO** | **NO** |  |
| **M179I** | **YES (+)** | **NO** | **NO** | **NO** |  |
| **T69L/S99N/M179I** | **YES (+)** | **YES** | **NO** | **NO** |  |
| **pMagFast T69L/S99N/N133K/M179I + nMagHigh M55A/T69L/S99N/N133K/ M179I** | **YES (++)** | **YES (++)** | **NO** | **NO** |  |
| **pMagFast T69L/S99N/N130K/N133K/ /M179I + nMagHigh M55A/T69L/ S99N/N130K/N133K/M179I** | **YES (-)** | **YES (-)** | **YES**  **(-)** | **NO** |  |
| **pMagFast T69L/S99N/N133F/M179I + nMagHigh M55A/T69L/S99N/ N133F/M179I** | **YES (++)** | **YES (++)** | **YES (++)** | **NO** | **Slower off kinetics (t_1/2_ = 1 min)** |
| **pMagFast T69L/S99N/N133L/M179I + nMagHigh M55A/T69L/S99N/N133L/M179I** | **YES (+)** | **YES (+)** | **YES (+)** | **NO** |  |
| **pMagFast T69L/S99N/D128A/N130 /N133K/M179I + nMagHigh M55A/ T69L/S99N/D128A/N133K/M179I** | **NO** | **NO** | **NO** | **NO** |  |
| **pMagFast T69L/S99N/D128E/N130 /N133K/M179I + nMagHigh M55A/ T69L/S99N/D128E/N133K/M179I** | **NO** | **NO** | **NO** | **NO** |  |
| **pMagFast T69L/S99N/N130Q /N133K/M179I + nMagHigh M55A/ T69L/S99N/N130Q /N133K/M179I** | **NO** | **NO** | **NO** | **NO** |  |
| **pMagFast T69L/S99N/K125R/N130 /M179I + nMagHigh M55A/ T69L/S99N/K125R/N130/M179I** | **NO** | **NO** | **NO** | **NO** |  |
| **pMagFast T69L/S99N/V103I/M179I + nMagHigh M55A/ T69L/S99N/V103I/M179I** | **NO** | **NO** | **NO** | **NO** |  |
| **pMagFast T69L/G94E/S99N/ N133F/M179I + nMagHigh M55A/T69L/G94E/S99N/ N133F/M179I** | **YES (+)** | **NT** | **NO** | **NO** | **Slower off kinetics** |
| **pMagFast T69L/S99N/ M117S/N133F/ /M179I + nMagHigh M55A/T69L/S99N/ M117S/N133F/ M179I** | **YES (+)** | **NT** | **NO** | **NO** |  |
| **pMagFast T69L/S99N/M117Y/ N133F/M179I + nMagHigh M55A/T69L/S99N/M117Y/ N133F/M179I** | **YES (+)** | **NT** | **NO** | **NO** |  |
| **pMagFast T69N/S99N/N133F/M179I +**  **nMagHigh M55A/T69N/S99N/N133F/M179I** | **YES (-)** | **NT** | **NO** | **NO** |  |
| **pMagFast T69L/S99N/N130K/N133F/ /M179I + nMagHigh M55A/T69L/S99N/N130K/N133F/ M179I** | **YES (+)** | **NT** | **NO** | **NO** | **Slower off kinetics** |
| **pMagFast T69L/S99N/N100D/ N133F/M179I + nMagHigh M55A/T69L/S99N/N100D/ N133F/M179I** | **YES (+)** | **NT** | **NO** | **NO** |  |
| **pMagFast T69L/S99N/K125R/ N133F/M179I + nMagHigh M55A/T69L/S99N/K125R/ N133F/M179I** | **YES (+)** | **NT** | **NO** | **NO** |  |
| **pMagFast T69L/Y94E/S99N/ N133F/M179I + nMagHigh M55A/T69L/Y94E/S99N/N133F/M179I** | **YES (+++)** | **NT** | **YES (+++)** | **YES (+)** |  |
| **pMagFast T69L/Y87F/Y94E/S99N/ N133F/M179I + nMagHigh M55A/T69L/Y87F/Y94E/S99N/ N133F/M179I** | **NO** | **NT** | **NO** | **NO** |  |
| **pMagFast T69I/Y94E/S99N/N133F/ /M179I + nMagHigh M55A/T69I/Y94E/S99N/N133F/M179I** | **YES (-)** | **NT** | **YES (-)** | **NO** |  |
| **pMagFast T69V/Y94E/S99N/N133F/ /M179I + nMagHigh M55A/ T69V/Y94E/S99N/N133F/M179I** | **NO** | **NT** | **NO** | **NO** |  |
| **pMagFast T69L/Y94E/S99N/N133Y/ /M179I + nMagHigh M55A/T69L/Y94E/S99N/ N133Y/M179I** | **YES (+++)** | **NT** | **YES (+++)** | **YES (++)** |  |
| **pMagFast T69L/Y94E/S99N/ N133F/S178C/M179I + nMagHigh M55A/T69L/Y94E/S99N/ N133F/S178C/M179I** | **YES (+)** | **NT** | **YES (+)** | **NO** |  |
| **pMagFast T69L/Y94E/S99N/ N133F/S178F/M179I + nMagHigh M55A/T69L/Y94E/S99N/ N133F/S178F/M179I** | **NO** | **NT** | **NO** | **NO** |  |
| **pMagFast T69L/Y94E/S99N/ N133F/I139L/M179I + nMagHigh M55A/T69L/Y94E/S99N/N133F/ I139L/M179I** | **NO** | **NT** | **NO** | **NO** |  |
| **pMagFast T69L/Y94E/S99N/ N133F/I139V/M179I + nMagHigh M55A/T69L/Y94E/S99N/N133F/ I139V/M179I** | **YES (+)** | **NT** | **YES (+)** | **NO** |  |
| **pMagFast T69L/Y94E/S99N/ N133F/R141E/M179I + nMagHigh M55A/T69L/Y94E/S99N/N133F/ R141E/M179I** | **YES (+++)** | **NT** | **YES (+++)** | **YES (++)** | **Slower off kinetics** |
| **pMagFast T69L/Y94E/S99N/ N133F/R136K/M179I + nMagHigh M55A/T69L/Y94E/S99N/N133F/ R136K/M179I** | **YES (+++)** | **NT** | **YES (+++)** | **YES (++)** |  |
| **pMagFast T69L/Y94E/S99N/N100R/ N133F/M179I + nMagHigh M55A/T69L/Y94E/S99N/N100R/ N133F/M179I** | **YES (+++)** | **NT** | **YES (+++)** | **YES (++)** |  |
| **pMagFast T69L/Y94E/S99N/N100R/A101H/ N133F/M179I + nMagHigh M55A/T69L/Y94E/S99N/N100R/ A101H/N133F/M179I** | **YES (+++)** | **NT** | **YES (+++)** | **YES (++)** |  |
| **pMagFast T69L/Y94E/S99N/M117V/ N133F/M179I + nMagHigh M55A/T69L/Y94E/S99N/M117V/ N133F/M179I** | **YES (++)** | **NT** | **YES (++)** | **YES (+)** |  |
| **pMagFast T69L/Y94E/S99N/Y126F/ N133F/M179I + nMagHigh M55A/T69L/Y94E/S99N/Y126F/ N133F/M179I** | **YES (+++)** | **NT** | **YES (+++)** | **YES (++)** |  |
| **pMagFast T69L/Y94E/S99N/N133F/ T134K/M179I + nMagHigh M55A/T69L/Y94E/S99N/N133F/ T134K/M179I** | **YES (++)** | **NT** | **YES (++)** | **NO** | **Slower off kinetics** |
| **pMagFast T69L/Y94E/S99N/N133F/ K137N/M179I + nMagHigh M55A/T69L/Y94E/S99N/N133F/ K137N/M179I** | **YES (++)** | **NT** | **YES (++)** | **NO** | **Slower off kinetics** |
| **pMagFast T69L/Y94E/S99N/N133F/ K137D/M179I + nMagHigh M55A/T69L/Y94E/S99N/N133F/ K137D/M179I** | **YES (++)** | **NT** | **YES (++)** | **YES(+)** |  |
| **pMagFast T69L/Y94E/S99N/N133F/ K153R/M179I + nMagHigh M55A/T69L/Y94E/S99N/N133F/ K153R/M179I** | **YES (++)** | **NT** | **YES (++)** | **NO** | **Slower off kinetics** |
| **pMagFast T69L/Y94E/S99N/V103I/ N133F/M179I + nMagHigh M55A/T69L/Y94E/S99N/V103I/N133F/ K153R/M179I** | **YES (++)** | **NT** | **YES (++)** | **NO** | **Slower off kinetics** |
| **eMags**  **pMagFast T69L/Y94E/S99N/N100R/A101H/ N133Y/R136K/M179I + nMagHigh M55A/T69L/Y94E/S99N/N100R/ A101H/N133Y/R136K/M179I** | **YES (+++)** | **NT** | **YES (+++)** | **YES (++++)** | **High dimerization efficiency at 37°C without any preincubation and faster on and off kinetics than original Magnets** |
| **eMags^F^**  **pMagFast T69L/Y94E/S99N/N100R/A101H/ N133F/R136K/M179I + nMagHigh M55A/T69L/Y94E/S99N/N100R/ A101H/N133F/R136K/M179I** | **YES (+++)** | **NT** | **YES (+++)** | **YES (+++)** | **Slightly lower dimerization efficiency than (eMags) but faster on and off kinetics** |
