# Supplementary material for: Optimized Vivid-derived Magnets photodimerizers for subcellular optogenetics in mammalian cells
Source: eLife. 2020 Nov 11;9:e63230. doi: 10.7554/eLife.63230 (PMC7735757; doi:10.7554/eLife.63230)
Supplement: Supplementary file 4. [file elife-63230-supp4.docx]

**PRIMERS DESIGN FOR OPTIMIZATION OF THE MAGNETS HETERODIMER INTERFACE**

**Different charge swaps**

**NEGATIVE MAGNET**

b. I52R/M55R & **I52E**/M55G

In the vector nMagHigh-EGFP-OMP25 (5486 bp) I will insert the point mutation I52E (I will convert aspartate D (GAT) with Glutamate E (GAA).

**Primers:**

**t876a AS** 5'-gggcggttcccaatctgttccaaatatcccataat-3'

**t876a** 5'-attatgggatatttggaacagattgggaaccgccc-3'

c. I52R/M55R & I52D/**M55A** (A should still leave enough room for the R and is a much better helix-former)

In the vector nMagHigh-EGFP-OMP25 (5486 bp) I will insert the point mutation M55A (I will convert glycine G (GGG) with Alanine A (GCG).

**Primers:**

**g884c AS** 5'-gtttgggcggttcgcaatctgatccaaatatccc-3'

**g884c**  5’-gggatatttggatcagattgcgaaccgcccaaac-3’

d. **M48R/M55R** & **M48E/M55E** should provide a double charge-pair for preventing homo- and favoring hetero-dimerization.

In the vector nMagHigh-EGFP-OMP25 (5486 bp) I will insert the point mutation M48E first (I will convert methionine M (ATG) with Glutamate E (GAG).

**a862g_t863a** 5'-cgcccctggaggatacgacattgagggatatttggatc-3'

**a862g_t863aAS** 5'-gatccaaatatccctcaatgtcgtatcctccaggggcg-3'

In the vector nMagHigh-EGFP-OMP25 (5486 bp) I will insert the point mutation M55E first (I will convert Glycine 55 (GGG) with Glutamate E (GAG).

**g884a AS** 5'-gtttgggcggttctcaatctgatccaaatatccc-3'

**g884a**_ 5'-gggatatttggatcagattgagaaccgcccaaac-3'

e. I52R/M55R & I52D/**M55E** (Has the best helix-forming propensity, could discourage homo-dimerization, and might provide another charge contact across the interface. It looks like there should be enough room to accommodate it.)

| **Primer Name** | **Primer Sequence (5' to 3')** |
| --- | --- |
|  | 5'-gtttgggcggttctcaatctgatccaaatatccc-3' |
| g884a_ | 5'-gggatatttggatcagattgagaaccgcccaaac-3' |

f. I52R/M55R & I52D/**M55D** (Might also be worth trying **M55D**, even though aspartate is not great at helices, but could give nice charge packing)

| **Primer Name** | **Primer Sequence (5' to 3')** |
| --- | --- |
|  | 5'-agggtttgggcggttgtcaatctgatccaaatatcccataa-3' |
| g884a_g885c_ | 5'-ttatgggatatttggatcagattgacaaccgcccaaaccct-3' |

**POSITIVE MAGNET**

d. **M48R/M55R** in the Positive Magnets & **M48E/M55E** should provide a double charge-pair for preventing homo- and favoring hetero-dimerization.

In the vector pMagFast3-tgRFPt (6593 bp) I will insert the point mutation M48R (I will convert Methionine 48 (ATG) with Arginine R (AGG).

**t963g AS** 5'-ctgaggtaacccctgatgtcataaccccccgg-3'

**t963g_** 5'-ccggggggttatgacatcaggggttacctcag-3'
