# Supplementary material for: Optimized Vivid-derived Magnets photodimerizers for subcellular optogenetics in mammalian cells
Source: eLife. 2020 Nov 11;9:e63230. doi: 10.7554/eLife.63230 (PMC7735757; doi:10.7554/eLife.63230)
Supplement: Supplementary file 5. [file elife-63230-supp5.docx]

**PRIMERS DESIGN FOR THERMOSTABILIZATION OF THE MAGNETS PROTEIN**

**1. Mito-nMagHigh-EGFP G49A/Y50F**

5'-aaatatcccataatgtcgaatgctccaggggcgtaaagag-3'

| g851c_a854t_ | 5'-ctctttacgcccctggagcattcgacattatgggatattt-3' |
| --- | --- |

**1. pMF-tgRFPt G49A/Y50F**

5'-taacccatgatgtcaaaagcccccggcgcgtag-3'

| g951c_a954t_ | 5'-ctacgcgccgggggcttttgacatcatgggtta-3' |
| --- | --- |

**2. Mito-nMagHigh-EGFP G49A/Y50I**

5'-tccaaatatcccataatgtcgattgctccaggggcgtaaagagtg-3'

g851c_t853a_a854t_ 5'-cactctttacgcccctggagcaatcgacattatgggatatttgga-3'

**2. pMF-tgRFPt G49A/Y50I**

5'-gtaacccatgatgtcaatagcccccggcgcgtagag-3'

g951c_t953a_a954t_ 5'-ctctacgcgccgggggctattgacatcatgggttac-3'

**3. Mito-nMagHigh-EGFP Y94E/N100E**

| 3A 5'-gccgagcacctctgcctcgctgtatccggtcat-3' |  |
| --- | --- |
| 3A t1000g_t1002a | 5'-gcctcggaagccttcttggaaatgaccggatacagcaat-3' |
| 3B t1000g_t1002a_antisense | 5'-attgctgtatccggtcatttccaagaaggcttccgaggc-3' |
| 3B a1018g_t1020g_ | 5'-atgaccggatacagcgaggcagaggtgctcggc-3' |

**3A. Mito-nMagHigh-EGFP Y94E**

| **Primer Name** | **Primer Sequence (5' to 3')** |
| --- | --- |
|  | 5'-attgctgtatccggtcatttccaagaaggcttccgaggc-3' |
| t999g_t1001a_ | 5'-gcctcggaagccttcttggaaatgaccggatacagcaat-3' |

**3. pMF-tgRFPt Y94E/N100E**

| 5'-cccagcacttcggcttccgagtacccggtca-3' |  |
| --- | --- |
| t1100g_c1102a | 5'-cctccgaagcattcctggaaatgaccgggtactcgaa-3' |
| t1100g_c1102a_antisense | 5'-ttcgagtacccggtcatttccaggaatgcttcggagg-3' |
| a1118g_c1120a_ | 5'-tgaccgggtactcggaagccgaagtgctggg-3' |

**4. Mito-nMagHigh-EGFP N130K/N133K**

|  | 5'-ccggtcgatggccttcttgatggtcttgatcgtgttcgagt-3' |
| --- | --- |
| c1119g_c1126a_g1127a_ | 5'-actcgaacacgatcaagaccatcaagaaggccatcgaccgg-3' |

**4 bis. Mito-nMagHigh-EGFP N130K in S99N N133K T69L I179M**

5'-tggtcttgatcgtcttcgagtccacatatttgcgag-3'

| c1109g_ | 5'-ctcgcaaatatgtggactcgaagacgatcaagacca-3' |
| --- | --- |

**4. pMF-tgRFPt N130K/N133K**

|  | 5'-gcggtcaatggccttcttcatggtcttgatagtgttggagtccacgtatttgc-3' |
| --- | --- |
| c1219g_c1226a_g1227a_c1228g_ | 5'-gcaaatacgtggactccaacactatcaagaccatgaagaaggccattgaccgc-3' |

**4 bis. pMF-tgRFPt N130K**

| **Primer Name** | **Primer Sequence (5' to 3')** |
| --- | --- |
|  | 5'-catggtcttgatagtcttggagtccacgtatttgc-3' |
| c1210g_ | 5'-gcaaatacgtggactccaagactatcaagaccatg-3' |

**5. Mito-nMagHigh-EGFP L64I/V67I/T69L**

| 5'ggatcagggcacatgacaggtcgataggcccgatttcgacctgagggtttgg-3' |  |
| --- | --- |
| c910a_g912c_g919a_g921c_a925c_c926t_ | 5'-ccaaaccctcaggtcgaaatcgggcctatcgacctgtcatgtgccctgatcc-3' |

**5. pMF-tgRFPt L64I/V67I/T69L**

|  | **Primer Sequence (5' to 3')** |
| --- | --- |
|  | 5'-gagggcgcaggagaggtcgatgggtccgatctccacttgtggg-3' |
| c1010a_g1012c_g1019a_a1025c_c1026t_ | 5'-cccacaagtggagatcggacccatcgacctctcctgcgccctc-3' |

**6. Mito-nMagHigh-EGFP L64I/V67I/T69M**

|  | **Primer Sequence (5' to 3')** |
| --- | --- |
|  | 5'-gatcagggcacatgacatgtcgataggcccgatttcgacctgagggtttg-3' |
| c910a_g912c_g919a_g921c_c926t_ | 5'-caaaccctcaggtcgaaatcgggcctatcgacatgtcatgtgccctgatc-3' |

**6. pMF-tgRFPt L64I/V67I/T69M**

|  | **Primer Sequence (5' to 3')** |
| --- | --- |
|  | 5'-cgagggcgcaggacatgtcgatgggtccgatctccacttgtggg-3' |
| c1010a_g1012c_g1019a_c1026t_c1027g_ | 5'-cccacaagtggagatcggacccatcgacatgtcctgcgccctcg-3' |

**7. Mito-nMagHigh-EGFP S99N/R106K**

|  | **Primer Sequence (5' to 3')** |
| --- | --- |
| **7A** | 5'-aggaatctgcagttcttgccgagcacctctg-3' |
| 7A g1016a | 5'-gtatatgaccggatacaacaatgcagaggtgctcg-3' |
| 7B g1016a_antisense | 5'-cgagcacctctgcattgttgtatccggtcatatac-3' |
| 7B g1037a_ | 5'-cagaggtgctcggcaagaactgcagattcct-3' |

**7A. Mito-nMagHigh-EGFP S99N**

| **Primer Name** | **Primer Sequence (5' to 3')** |
| --- | --- |
|  | 5'-cgagcacctctgcattgttgtatccggtcatatac-3' |
| g1015a_ | 5'-gtatatgaccggatacaacaatgcagaggtgctcg-3' |

**7. pMF-tgRFPt S99N/R106K**

|  | **Primer Sequence (5' to 3')** |
| --- | --- |
| **7A** | 5'-aggaagcggcagttctttcccagcacttcggc-3' |
| **7A** c1136a_g1137a_ | 5'-gccgaagtgctgggaaagaactgccgcttcct-3' |
| 7B t1115a_c1116a_g1117c | 5'-tgtacatgaccgggtacaacaacgccgaagtgctggg-3' |
| 7B t1115a_c1116a_g1117c_antisense | 5'-cccagcacttcggcgttgttgtacccggtcatgtaca-3' |

**8. Mito-nMagHigh-EGFP T123R/K125R/D128R/N130D**

|  | **Primer Sequence (5' to 3')** |
| --- | --- |
|  | 5'-gatggtgttgatcgtgtccgaggccacatatctgcgtctcgactttggtttcaccat-3' |
| c1088g_t1089a_a1094g_a1103c_a1108g_ | 5'-atggtgaaaccaaagtcgagacgcagatatgtggcctcggacacgatcaacaccatc-3' |

**8. pMF-tgRFPt T123R/K125R/D128R/N130D**

|  | **Primer Sequence (5' to 3')** |
| --- | --- |
|  | 5'-catggtgttgatagtgtcggaggccacgtatctgcgtcttgacttaggcttcaccat-3' |
| c1188g_c1189a_a1194g_a1203c_a1208g_ | 5'-atggtgaagcctaagtcaagacgcagatacgtggcctccgacactatcaacaccatg-3' |

**9. Mito-nMagHigh-EGFP N56T**

| 5'-gggtttgggcgggtcccaatctgatccaaatatccc-3' |  |
| --- | --- |
| a886c_ | 5'-gggatatttggatcagattgggacccgcccaaaccc-3' |

**9. pMF-tgRFPt N56T**

| **Primer Name** | **Primer Sequence (5' to 3')** |
| --- | --- |
|  | 5'-ggttcggccgggttctgatctgtctgaggtaac-3' |
| a987c_ | 5'-gttacctcagacagatcagaacccggccgaacc-3' |

**10. Mito-nMagHigh-EGFP L64I**

| **Primer Name** | **Primer Sequence (5' to 3')** |
| --- | --- |
|  | 5'-tccacaggccctatttcgacctgagggtttgggc-3' |
| c909a_g911a_ | 5'-gcccaaaccctcaggtcgaaatagggcctgtgga-3' |

**10. pMF-tgRFPt L64I**

| **Primer Name** | **Primer Sequence (5' to 3')** |
| --- | --- |
|  | 5'-ggtgtcgacgggtcctatctccacttgtgggtt-3' |
| c1010a_g1012a_ | 5'-aacccacaagtggagataggacccgtcgacacc-3' |

**11. Mito-nMagHigh-EGFP V67I**

| **Primer Name** | **Primer Sequence (5' to 3')** |
| --- | --- |
|  | 5'-ggcacatgacgtgtctataggccccagttcgac-3' |
| g918a_g920a_ | 5'-gtcgaactggggcctatagacacgtcatgtgcc-3' |

**11. pMF-tgRFPt V67I**

| **Primer Name** | **Primer Sequence (5' to 3')** |
| --- | --- |
|  | 5'-cgcaggaggtgtctatgggtcccagctcc-3' |
| g1019a_c1021a_ | 5'-ggagctgggacccatagacacctcctgcg-3' |

**12. Mito-nMagHigh-EGFP T69L**

| **Primer Name** | **Primer Sequence (5' to 3')** |
| --- | --- |
|  | 5'-atcagggcacatgacaggtccacaggccccag-3' |
| a924c_c925t_ | 5'-ctggggcctgtggacctgtcatgtgccctgat-3' |

**12. pMF-tgRFPt T69L**

| **Primer Name** | **Primer Sequence (5' to 3')** |
| --- | --- |
|  | 5'-gagggcgcaggagaggtcgacgggtccc-3' |
| a1025c_c1026t_ | 5'-gggacccgtcgacctctcctgcgccctc-3' |

**13. Mito-nMagHigh-EGFP F162I**

| **Primer Name** | **Primer Sequence (5' to 3')** |
| --- | --- |
|  | 5'-ggaatgatggtcagaatgttcacgaaccgctgg-3' |
| t1203a_ | 5'-ccagcggttcgtgaacattctgaccatcattcc-3' |

**13. pMF-tgRFPt F162I**

| **Primer Name** | **Primer Sequence (5' to 3')** |
| --- | --- |
| t1304a_ | 5'-gacagcgcttcgtcaacatcctgactatgattccc-3' |
|  | 5'-gggaatcatagtcaggatgttgacgaagcgctgtc-3' |

**14. Mito-nMagHigh-EGFP F162L**

| **Primer Name** | **Primer Sequence (5' to 3')** |
| --- | --- |
|  | 5'-ggaatgatggtcagaaggttcacgaaccgctgg-3' |
| t1203c_ | 5'-ccagcggttcgtgaaccttctgaccatcattcc-3' |

**14. pMF-tgRFPt F162L**

| **Primer Name** | **Primer Sequence (5' to 3')** |
| --- | --- |
|  | 5'-gggaatcatagtcaggaggttgacgaagcgctgtc-3' |
| t1304c_ | 5'-gacagcgcttcgtcaacctcctgactatgattccc-3' |

**15. Mito-nMagHigh-EGFP M179I**

| **Primer Name** | **Primer Sequence (5' to 3')** |
| --- | --- |
|  | 5'-cgcactggaatccgatggagtatctgtactctcc-3' |
| g1256c_ | 5'-ggagagtacagatactccatcggattccagtgcg-3' |

**15. pMF-tgRFPt M179I**

| **Primer Name** | **Primer Sequence (5' to 3')** |
| --- | --- |
|  | 5'-cgcactgaaacccgatgctgtaccggtattcg-3' |
| g1357c_ | 5'-cgaataccggtacagcatcgggtttcagtgcg-3' |

**SECOND ROUND OF SINGLE MUTATIONS AND FIRST COMBINATIONS:**

1. pMagFast-tgRFPt number **4** as template (I52R/M55R and N130K/N133K)

We should add **S99N**

| **Primer Name** | **Primer Sequence (5' to 3')** |
| --- | --- |
|  | 5'-cccagcacttcggcgttgttgtacccggtcatgtaca-3' |
| t1115a_c1116a_g1117c_ | 5'-tgtacatgaccgggtacaacaacgccgaagtgctggg-3' |

2. nMagHigh-EGFP-OMP25 number **4** as template (N130K/N133K)

We should add **S99N** first

| **Primer Name** | **Primer Sequence (5' to 3')** |
| --- | --- |
|  | 5'-cgagcacctctgcattgttgtatccggtcatatac-3' |
| g1016a_ | 5'-gtatatgaccggatacaacaatgcagaggtgctcg-3' |

Then charge swap: **M55A**

| **Primer Name** | **Primer Sequence (5' to 3')** |
| --- | --- |
|  | 5'-gtttgggcggttcgcaatctgatccaaatatccc-3' |
| g883c_ | 5'-gggatatttggatcagattgcgaaccgcccaaac-3' |

**THIRD ROUND OF IMPROVEMENT:**

**3.1. Starting from nMagHigh M55A/ T69L/S99N/N130 /N133K**

**3.1** and instead of making N133K, put **N133L**

AS 5'-atggccttcttgatggtcaggatcgtgttcgagtccac-3'

| a1116c_a1117t_ | 5'-gtggactcgaacacgatcctgaccatcaagaaggccat-3' |
| --- | --- |

**3.2** and instead of making N133K, put **N133F**

| **Primer Name** | **Primer Sequence (5' to 3')** |
| --- | --- |
|  | 5'-cgatggccttcttgatggtgaagatcgtgttcgagtccaca-3' |
| a1116t_a1117t_g1118c_ | 5'-tgtggactcgaacacgatcttcaccatcaagaaggccatcg-3' |

**3.1. Starting from** pMagFast T69L/ S99N/**N130** /N133K/M179I

**3.1** and instead of making N133K, put **N133L**

| **Primer Name** | **Primer Sequence (5' to 3')** |
| --- | --- |
|  | 5'-aatggccttcttcatggtcaggatagtgttggagtccacg-3' |
| a1217c_a1218t_ | 5'-cgtggactccaacactatcctgaccatgaagaaggccatt-3' |

**3.2** and instead of making N133K, put **N133F**

| **Primer Name** | **Primer Sequence (5' to 3')** |
| --- | --- |
|  | 5'-gtcaatggccttcttcatggtgaagatagtgttggagtccacgta-3' |
| a1217t_a1218t_g1219c_ | 5'-tacgtggactccaacactatcttcaccatgaagaaggccattgac-3' |

**4.1. Starting from nMagHigh M55A/ T69L/S99N/N130 /N133K**

**4.1** **D128E**

| **Primer Name** | **Primer Sequence (5' to 3')** |
| --- | --- |
|  | 5'-cttgatcgtgttcgattccacatatttgcgagtcgac-3' |
| c1103a_ | 5'-gtcgactcgcaaatatgtggaatcgaacacgatcaag-3' |

**4.2** **D128A**

| **Primer Name** | **Primer Sequence (5' to 3')** |
| --- | --- |
|  | 5'-tcttgatcgtgttcgaggccacatatttgcgagtc-3' |
| a1102c_ | 5'-gactcgcaaatatgtggcctcgaacacgatcaaga-3' |

**4.2** **D128A + N130D**

| **Primer Name** | **Primer Sequence (5' to 3')** |
| --- | --- |
|  | 5'-gatggtcttgatcgtgtccgaggccacatatttgc-3' |
| a1107g_ | 5'-gcaaatatgtggcctcggacacgatcaagaccatc-3' |

**4.1. Starting from** pMagFast T69L/ S99N/**N130** /N133K/M179I

**4.1** **D128E**

| **Primer Name** | **Primer Sequence (5' to 3')** |
| --- | --- |
|  | 5'-cttgatagtgttggattccacgtatttgcgggttgac-3' |
| c1204a_ | 5'-gtcaacccgcaaatacgtggaatccaacactatcaag-3' |

**4.2** **D128A -> If this mutation works, combo with N130D**

| **Primer Name** | **Primer Sequence (5' to 3')** |
| --- | --- |
|  | 5'-tcttgatagtgttggaggccacgtatttgcgggtt-3' |
| a1203c_ | 5'-aacccgcaaatacgtggcctccaacactatcaaga-3' |

**4.2** **D128A + N130D**

| **Primer Name** | **Primer Sequence (5' to 3')** |
| --- | --- |
|  | 5'-catggtcttgatagtgtcggaggccacgtatttgc-3' |
| a1208g_ | 5'-gcaaatacgtggcctccgacactatcaagaccatg-3' |

**5.1. Starting from** pMagFast T69L/ S99N/**N130** /N133K/M179I

N130Q

| **Primer Name** | **Primer Sequence (5' to 3')** |
| --- | --- |
|  | 5'-tcttcatggtcttgatagtctgggagtccacgtatttgcgg-3' |
| a1208c_c1210g_ | 5'-ccgcaaatacgtggactcccagactatcaagaccatgaaga-3' |

**6.1. Starting from** pMagFast T69L/ S99N/**N130** /N133K/M179I

**K125R**

| **Primer Name** | **Primer Sequence (5' to 3')** |
| --- | --- |
|  | 5'-gtgttggagtccacgtacctgcgggttgacttaggc-3' |
| a1194g_a1195g_ | 5'-gcctaagtcaacccgcaggtacgtggactccaacac-3' |

**7.1. Starting from** pMagFast T69L/ S99N/**N130** /N133K/M179I

**V103I**

| **Primer Name** | **Primer Sequence (5' to 3')** |
| --- | --- |
|  | 5'-ttccgtcccagtatttcggcgttcgagtacccgg-3' |
| g1127a_g1129a_ | 5'-ccgggtactcgaacgccgaaatactgggacggaa-3' |

S99L

| **Primer Name** | **Primer Sequence (5' to 3')** |
| --- | --- |
|  | 5'-ttccgtcccagtatttcggcgttgttgtacccgg-3' |
| g1127a_g1129a_ | 5'-ccgggtacaacaacgccgaaatactgggacggaa-3' |

**5.1. Starting from nMagHigh M55A/ T69L/S99N/N130 /N133K**

N130Q

| **Primer Name** | **Primer Sequence (5' to 3')** |
| --- | --- |
|  | 5'-tcttgatggtcttgatcgtctgcgagtccacatatttgcga-3' |
| a1107c_c1109g_ | 5'-tcgcaaatatgtggactcgcagacgatcaagaccatcaaga-3' |

**6.1. Starting from nMagHigh M55A/ T69L/S99N/N130 /N133K**

K125R

| **Primer Name** | **Primer Sequence (5' to 3')** |
| --- | --- |
|  | 5'-tcgtgttcgagtccacatacctgcgagtcgactttggttt-3' |
| a1093g_a1094g_ | 5'-aaaccaaagtcgactcgcaggtatgtggactcgaacacga-3' |

**7.1. Starting from nMagHigh M55A/ T69L/S99N/N130 /N133K**

V103I

| **Primer Name** | **Primer Sequence (5' to 3')** |
| --- | --- |
|  | 5'-tctgcagttcctgccgagtatctctgcattgttgtatcc-3' |
| g1026a_g1028a_ | 5'-ggatacaacaatgcagagatactcggcaggaactgcaga-3' |

**5.1. Starting from pMF and nMH thermostable at 35C**

**5.1. Starting from** pMagFast T69L/ S99N/**N130F** /N133K/M179I

1. G49A

| **Primer Name** | **Primer Sequence (5' to 3')** |
| --- | --- |
|  | 5'-tgatctgtctgaggtaagccatgatgtcataaccc-3' |
| g966c_ | 5'-gggttatgacatcatggcttacctcagacagatca-3' |

2. M117S

| **Primer Name** | **Primer Sequence (5' to 3')** |
| --- | --- |
|  | 5'-gttgacttaggcttcacgcttccatccggggattgca-3' |
| t1170g_g1171c_ | 5'-tgcaatccccggatggaagcgtgaagcctaagtcaac-3' |

3. M117Y

| **Primer Name** | **Primer Sequence (5' to 3')** |
| --- | --- |
|  | 5'-ggttgacttaggcttcacgtatccatccggggattgcag-3' |
| a1169t_t1170a_g1171c_ | 5'-ctgcaatccccggatggatacgtgaagcctaagtcaacc-3' |

4. N100D

| **Primer Name** | **Primer Sequence (5' to 3')** |
| --- | --- |
|  | 5'-cagcacttcggcgtcgttgtacccggtcat-3' |
| a1118g_ | 5'-atgaccgggtacaacgacgccgaagtgctg-3' |

5. Y94E

| **Primer Name** | **Primer Sequence (5' to 3')** |
| --- | --- |
|  | 5'-ttgttgtacccggtcatctccaggaatgcttcggagg-3' |
| t1100g_c1102g_ | 5'-cctccgaagcattcctggagatgaccgggtacaacaa-3' |

6. N130K

| **Primer Name** | **Primer Sequence (5' to 3')** |
| --- | --- |
|  | 5'-catggtgaagatagtcttggagtccacgtatttgc-3' |
| c1210g_ | 5'-gcaaatacgtggactccaagactatcttcaccatg-3' |

7. T69N

| **Primer Name** | **Primer Sequence (5' to 3')** |
| --- | --- |
|  | 5'-gagggcgcaggagttgtcgacgggtccc-3' |
| c1025a_t1026a_ | 5'-gggacccgtcgacaactcctgcgccctc-3' |

8. K125R

| **Primer Name** | **Primer Sequence (5' to 3')** |
| --- | --- |
|  | 5'-gtgttggagtccacgtatcggcgggttgacttaggctt-3' |
| a1193c_a1194g_ | 5'-aagcctaagtcaacccgccgatacgtggactccaacac-3' |

9. M117S

| **Primer Name** | **Primer Sequence (5' to 3')** |
| --- | --- |
|  | 5'-gttgacttaggcttcacgcttccatccggggattgca-3' |
| t1170g_g1171c_ | 5'-tgcaatccccggatggaagcgtgaagcctaagtcaac-3' |

**5.1. Starting from nMagHigh M55A/ T69L/S99N/N130F /N133K**

1. G49A

| **Primer Name** | **Primer Sequence (5' to 3')** |
| --- | --- |
|  | 5'-cgcaatctgatccaaatatgccataatgtcgtatcctcc-3' |
| g865c_ | 5'-ggaggatacgacattatggcatatttggatcagattgcg-3' |

2. M117S

| **Primer Name** | **Primer Sequence (5' to 3')** |
| --- | --- |
| t1069g_g1070c_ | 5'-gtcccccgacgggagcgtgaaaccaaagtcg-3' |
|  | 5'-cgactttggtttcacgctcccgtcgggggac-3' |

3. M117Y

| **Primer Name** | **Primer Sequence (5' to 3')** |
| --- | --- |
| a1068t_t1069a_g1070c_ | 5'-gcagtcccccgacgggtacgtgaaaccaaagtcgac-3' |
|  | 5'-gtcgactttggtttcacgtacccgtcgggggactgc-3' |

4. N100D

| **Primer Name** | **Primer Sequence (5' to 3')** |
| --- | --- |
|  | 5'-ccgagcacctctgcatcgttgtatccggtcata-3' |
| a1017g_ | 5'-tatgaccggatacaacgatgcagaggtgctcgg-3' |

5. Y94E

| **Primer Name** | **Primer Sequence (5' to 3')** |
| --- | --- |
|  | 5'-cattgttgtatccggtcatctccaagaaggcttccgaggcg-3' |
| t999g_t1001g_ | 5'-cgcctcggaagccttcttggagatgaccggatacaacaatg-3' |

6. N130K

| **Primer Name** | **Primer Sequence (5' to 3')** |
| --- | --- |
|  | 5'-tggtgaagatcgtcttcgagtccacatatttgcgag-3' |
| c1109g_ | 5'-ctcgcaaatatgtggactcgaagacgatcttcacca-3' |

7. T69N

| **Primer Name** | **Primer Sequence (5' to 3')** |
| --- | --- |
|  | 5'-ggatcagggcacatgagttgtccacaggccccagt-3' |
| c924a_t925a_g926c_ | 5'-actggggcctgtggacaactcatgtgccctgatcc-3' |

8. K125R

| **Primer Name** | **Primer Sequence (5' to 3')** |
| --- | --- |
|  | 5'-cgtgttcgagtccacatatcggcgagtcgactttggtttc-3' |
| a1092c_a1093g_ | 5'-gaaaccaaagtcgactcgccgatatgtggactcgaacacg-3' |

9. M117S

| **Primer Name** | **Primer Sequence (5' to 3')** |
| --- | --- |
| t1069g_g1070c_ | 5'-gtcccccgacgggagcgtgaaaccaaagtcg-3' |
|  | 5'-cgactttggtttcacgctcccgtcgggggac-3' |

**6.1. Starting from** pMagFast T69L/ **Y94E**/S99N/**N130F** /N133K/M179I

**1. Y87F**

| **Primer Name** | **Primer Sequence (5' to 3')** |
| --- | --- |
|  | 5'-tcggaggcgaacaccacaggggtgtcc-3' |
| a1080t_ | 5'-ggacacccctgtggtgttcgcctccga-3' |

**2. T69I**

| **Primer Name** | **Primer Sequence (5' to 3')** |
| --- | --- |
|  | 5'-gggcgcaggagatgtcgacgggtcc-3' |
| c1025a_ | 5'-ggacccgtcgacatctcctgcgccc-3' |

**3. T69V**

| **Primer Name** | **Primer Sequence (5' to 3')** |
| --- | --- |
|  | 5'-ggcgcaggagacgtcgacgggtc-3' |
| c1025g_ | 5'-gacccgtcgacgtctcctgcgcc-3' |

**4. N133Y**

| **Primer Name** | **Primer Sequence (5' to 3')** |
| --- | --- |
|  | 5'-tggccttcttcatggtgtagatagtgttggagtcc-3' |
| t1218a_ | 5'-ggactccaacactatctacaccatgaagaaggcca-3' |

**5. S178C**

| **Primer Name** | **Primer Sequence (5' to 3')** |
| --- | --- |
|  | 5'-actgaaacccgatgcagtaccggtattcgcc-3' |
| a1352t_ | 5'-ggcgaataccggtactgcatcgggtttcagt-3' |

**6. S178F**

| **Primer Name** | **Primer Sequence (5' to 3')** |
| --- | --- |
|  | 5'-actgaaacccgatgcagtaccggtattcgcc-3' |
| a1352t_g1353t | 5'-ggcgaataccggtacttcatcgggtttcagt-3' |

**7. I139L**

| **Primer Name** | **Primer Sequence (5' to 3')** |
| --- | --- |
|  | 5'-cctcagcattgcggtctaaggccttcttcatggtg-3' |
| a1235t_t1237a_ | 5'-caccatgaagaaggccttagaccgcaatgctgagg-3' |

**8. I139V**

| **Primer Name** | **Primer Sequence (5' to 3')** |
| --- | --- |
|  | 5'-tcagcattgcggtcaacggccttcttcatggtg-3' |
| a1235g_ | 5'-caccatgaagaaggccgttgaccgcaatgctga-3' |

**9. R141E**

| **Primer Name** | **Primer Sequence (5' to 3')** |
| --- | --- |
|  | 5'-ccacttgcacctcagcattctcgtcaatggccttcttcatg-3' |
| c1241g_g1242a_c1243g_ | 5'-catgaagaaggccattgacgagaatgctgaggtgcaagtgg-3' |

**10. N100R**

| **Primer Name** | **Primer Sequence (5' to 3')** |
| --- | --- |
|  | 5'-cagcacttcggctctgttgtacccggtcatctccagg-3' |
| a1119g_c1120a_ | 5'-cctggagatgaccgggtacaacagagccgaagtgctg-3' |

**11. A101H**

| **Primer Name** | **Primer Sequence (5' to 3')** |
| --- | --- |
| g1121c_c1122a_ | 5'-accgggtacaacaaccacgaagtgctgggacg-3' |
|  | 5'-cgtcccagcacttcgtggttgttgtacccggt-3' |

**12. N100R/A101H**

| **Primer Name** | **Primer Sequence (5' to 3')** |
| --- | --- |
|  | 5'-ccgtcccagcacttcgtgtctgttgtacccggtcatctcca-3' |
| a1119g_c1120a_g1121c_c1122a_ | 5'-tggagatgaccgggtacaacagacacgaagtgctgggacgg-3' |

**13. M117V**

| **Primer Name** | **Primer Sequence (5' to 3')** |
| --- | --- |
|  | 5'-taggcttcaccactccatccggggattgcag-3' |
| a1169g_ | 5'-ctgcaatccccggatggagtggtgaagccta-3' |

**14. Y126F**

| **Primer Name** | **Primer Sequence (5' to 3')** |
| --- | --- |
|  | 5'-tgttggagtccacgaatttgcgggttgacttagg-3' |
| a1197t_ | 5'-cctaagtcaacccgcaaattcgtggactccaaca-3' |

**15. T134K**

| **Primer Name** | **Primer Sequence (5' to 3')** |
| --- | --- |
|  | 5'-cggtcaatggccttcttcattttgaagatagtgttggagtcc-3' |
| c1221a_c1222a_ | 5'-ggactccaacactatcttcaaaatgaagaaggccattgaccg-3' |

**16. K137N**

| **Primer Name** | **Primer Sequence (5' to 3')** |
| --- | --- |
|  | 5'-gcggtcaatggcattcttcatggtgaagatagtgtt-3' |
| g1231t_ | 5'-aacactatcttcaccatgaagaatgccattgaccgc-3' |

**17. K137D**

| **Primer Name** | **Primer Sequence (5' to 3')** |
| --- | --- |
|  | 5'-cagcattgcggtcaatggcatccttcatggtgaagatagtg-3' |
| a1229g_g1231t_ | 5'-cactatcttcaccatgaaggatgccattgaccgcaatgctg-3' |

**18. K153R**

| **Primer Name** | **Primer Sequence (5' to 3')** |
| --- | --- |
|  | 5'-cgaagcgctgtccgttctttctgaagttcaccacttccac-3' |
| a1278g_g1279a_ | 5'-gtggaagtggtgaacttcagaaagaacggacagcgcttcg-3' |

**19. V103I**

| **Primer Name** | **Primer Sequence (5' to 3')** |
| --- | --- |
|  | 5'-ttccgtcccagtatttcggcgttgttgtacccgg-3' |
| g1127a_g1129a_ | 5'-ccgggtacaacaacgccgaaatactgggacggaa-3' |

**6.1. Starting from nMagHigh M55A/ T69L/Y94E/ S99N/N130F /N133K**

**1. Y87F**

| **Primer Name** | **Primer Sequence (5' to 3')** |
| --- | --- |
|  | 5'-tccgaggcgaagacgatcggagtgtcc-3' |
| a979t_ | 5'-ggacactccgatcgtcttcgcctcgga-3' |

**2. T69I**

| **Primer Name** | **Primer Sequence (5' to 3')** |
| --- | --- |
|  | 5'-gatcagggcacatgagatgtccacaggccccag-3' |
| c924a_g926c_ | 5'-ctggggcctgtggacatctcatgtgccctgatc-3' |

**3. T69V**

| **Primer Name** | **Primer Sequence (5' to 3')** |
| --- | --- |
|  | 5'-gggcacatgacacgtccacaggccc-3' |
| c924g_ | 5'-gggcctgtggacgtgtcatgtgccc-3' |

**4. N133Y**

| **Primer Name** | **Primer Sequence (5' to 3')** |
| --- | --- |
| t1117a_ | 5'-gactcgaacacgatctacaccatcaagaaggcc-3' |
|  | 5'-ggccttcttgatggtgtagatcgtgttcgagtc-3' |

**5. S178C**

| **Primer Name** | **Primer Sequence (5' to 3')** |
| --- | --- |
|  | 5'-gcactggaatccgatgcagtatctgtactctcc-3' |
| c1252g_ | 5'-ggagagtacagatactgcatcggattccagtgc-3' |

**6. S178F**

| **Primer Name** | **Primer Sequence (5' to 3')** |
| --- | --- |
|  | 5'-cgcactggaatccgatgaagtatctgtactctccg-3' |
| c1252t_ | 5'-cggagagtacagatacttcatcggattccagtgcg-3' |

**7. I139L**

| **Primer Name** | **Primer Sequence (5' to 3')** |
| --- | --- |
|  | 5'-ggcgttccggtcgagggccttcttgatgg-3' |
| a1134c_ | 5'-ccatcaagaaggccctcgaccggaacgcc-3' |

**8. I139V**

| **Primer Name** | **Primer Sequence (5' to 3')** |
| --- | --- |
|  | 5'-ggcgttccggtcgacggccttcttgatgg-3' |
| a1134g_ | 5'-ccatcaagaaggccgtcgaccggaacgcc-3' |

**9. R141E**

| **Primer Name** | **Primer Sequence (5' to 3')** |
| --- | --- |
|  | 5'-ggacctcggcgttctcgtcgatggccttct-3' |
| c1140g_g1141a_ | 5'-agaaggccatcgacgagaacgccgaggtcc-3' |

**10. N100R**

| **Primer Name** | **Primer Sequence (5' to 3')** |
| --- | --- |
|  | 5'-gagcacctctgctctgttgtatccggtcatctccaag-3' |
| a1018g_t1019a_ | 5'-cttggagatgaccggatacaacagagcagaggtgctc-3' |

**11. A101H**

| **Primer Name** | **Primer Sequence (5' to 3')** |
| --- | --- |
|  | 5'-ctgccgagcacctcatgattgttgtatccggtcatctccaaga-3' |
| g1020c_c1021a_a1022t_ | 5'-tcttggagatgaccggatacaacaatcatgaggtgctcggcag-3' |

**12. N100R/A101H**

| **Primer Name** | **Primer Sequence (5' to 3')** |
| --- | --- |
|  | 5'-gcagttcctgccgagcacctcatgtctgttgtatccggtcatctcca-3' |
| a1018g_t1019a_g1020c_c1021a_a1022t_ | 5'-tggagatgaccggatacaacagacatgaggtgctcggcaggaactgc-3' |

5'-tggagatgaccggatacaacagacatgaggtgctcggcaggaactgc-3'

**13. M117V**

| **Primer Name** | **Primer Sequence (5' to 3')** |
| --- | --- |
|  | 5'-ctttggtttcaccaccccgtcgggggact-3' |
| a1068g_ | 5'-agtcccccgacggggtggtgaaaccaaag-3' |

**14. Y126F**

| **Primer Name** | **Primer Sequence (5' to 3')** |
| --- | --- |
|  | 5'-tgttcgagtccacaaatttgcgagtcgactttgg-3' |
| a1096t_ | 5'-ccaaagtcgactcgcaaatttgtggactcgaaca-3' |

**15. T134K**

| **Primer Name** | **Primer Sequence (5' to 3')** |
| --- | --- |
|  | 5'-ggtcgatggccttcttgattttgaagatcgtgttcgagtc-3' |
| c1120a_c1121a_ | 5'-gactcgaacacgatcttcaaaatcaagaaggccatcgacc-3' |

**16. K137N**

| **Primer Name** | **Primer Sequence (5' to 3')** |
| --- | --- |
|  | 5'-cggtcgatggcattcttgatggtgaagatcgtg-3' |
| g1130t_ | 5'-cacgatcttcaccatcaagaatgccatcgaccg-3' |

**17. K137D**

| **Primer Name** | **Primer Sequence (5' to 3')** |
| --- | --- |
|  | 5'-gcgttccggtcgatggcatccttgatggtgaagatcg-3' |
| a1128g_g1130t_ | 5'-cgatcttcaccatcaaggatgccatcgaccggaacgc-3' |

**18. K153R**

| **Primer Name** | **Primer Sequence (5' to 3')** |
| --- | --- |
|  | 5'-gctggccgttctttctaaagttgaccacctccacctgga-3' |
| a1177g_g1178a_ | 5'-tccaggtggaggtggtcaactttagaaagaacggccagc-3' |

**19. V103I**

| **Primer Name** | **Primer Sequence (5' to 3')** |
| --- | --- |
|  | 5'-tctgcagttcctgccgagtatctctgcattgttgtatcc-3' |
| g1026a_g1028a_ | 5'-ggatacaacaatgcagagatactcggcaggaactgcaga-3' |
