# Supplementary material for: Optimized Vivid-derived Magnets photodimerizers for subcellular optogenetics in mammalian cells
Source: eLife. 2020 Nov 11;9:e63230. doi: 10.7554/eLife.63230 (PMC7735757; doi:10.7554/eLife.63230)
Supplement: Supplementary file 6. [file elife-63230-supp6.docx]

**Supplementary File 6: Fit parameters**

| Method | NonLinearLeastSquares |
| --- | --- |
| Robust | Off |
| Algorithm | Trust-Region |
| DiffMinChange | 1.00E-08 |
| DiffMaxChange | 0.1 |
| MaxFunEvals | 600 |
| MaxIter | 400 |
| TolFun | 1.00E-06 |
| TolX | 1.00E-06 |
