# Supplementary material for: Optimized Vivid-derived Magnets photodimerizers for subcellular optogenetics in mammalian cells
Source: eLife. 2020 Nov 11;9:e63230. doi: 10.7554/eLife.63230 (PMC7735757; doi:10.7554/eLife.63230)
Supplement: Transparent reporting form [file elife-63230-transrepform.docx]

***eLife’s* transparent reporting form**

We encourage authors to provide detailed information *within their submission* to facilitate the interpretation and replication of experiments. Authors can upload supporting documentation to indicate the use of appropriate reporting guidelines for health-related research (see [EQUATOR Network](http://www.equator-network.org/%20)), life science research (see the [BioSharing Information Resource](https://biosharing.org/)), or the [ARRIVE guidelines](http://www.plosbiology.org/article/info:doi/10.1371/journal.pbio.1000412) for reporting work involving animal research. Where applicable, authors should refer to any relevant reporting standards documents in this form.

If you have any questions, please consult our Journal Policies and/or contact us: [editorial@elifesciences.org](mailto:editorial@elifesciences.org).

**Sample-size estimation**

- You should state whether an appropriate sample size was computed when the study was being designed
- You should state the statistical method of sample size computation and any required assumptions
- If no explicit power analysis was used, you should describe how you decided what sample (replicate) size (number) to use

Please outline where this information can be found within the submission (e.g., sections or figure legends), or explain why this information doesn’t apply to your submission:

Sample size computation and power analysis were not performed. Since there was no prior expectation of effect size, we did not use any statistical methods to estimate sample size. For all experiments, data were obtained from multiple samples from at least two independent experiments or technical replicates to ensure robustness.

**Replicates**

- You should report how often each experiment was performed
- You should include a definition of biological versus technical replication
- The data obtained should be provided and sufficient information should be provided to indicate the number of independent biological and/or technical replicates
- If you encountered any outliers, you should describe how these were handled
- Criteria for exclusion/inclusion of data should be clearly stated
- High-throughput sequence data should be uploaded before submission, with a private link for reviewers provided (these are available from both GEO and ArrayExpress)

Please outline where this information can be found within the submission (e.g., sections or figure legends), or explain why this information doesn’t apply to your submission:

The number of cells analyzed in every experiment (biological replicates, N) and the total number of independent experiments (technical replicates) is reported in the figure legends and/or in the main text. No outliers where excluded.

**Statistical reporting**

- Statistical analysis methods should be described and justified
- Raw data should be presented in figures whenever informative to do so (typically when N per group is less than 10)
- For each experiment, you should identify the statistical tests used, exact values of N, definitions of center, methods of multiple test correction, and dispersion and precision measures (e.g., mean, median, SD, SEM, confidence intervals; and, for the major substantive results, a measure of effect size (e.g., Pearson's r, Cohen's d)
- Report exact p-values wherever possible alongside the summary statistics and 95% confidence intervals. These should be reported for all key questions and not only when the p-value is less than 0.05.

Please outline where this information can be found within the submission (e.g., sections or figure legends), or explain why this information doesn’t apply to your submission:

Results are reported as mean ± SEM. The most appropriate statistical analysis was performed for each data set according to sample size and distribution. The details on the statistical analysis performed are described in the results and materials and methods section (Image Analysis and Statistics paragraph). When informative, single data points are included in the figures (Fig. 1D).

(For large datasets, or papers with a very large number of statistical tests, you may upload a single table file with tests, Ns, etc., with reference to sections in the manuscript.)

**Group allocation**

- Indicate how samples were allocated into experimental groups (in the case of clinical studies, please specify allocation to treatment method); if randomization was used, please also state if restricted randomization was applied
- Indicate if masking was used during group allocation, data collection and/or data analysis

Please outline where this information can be found within the submission (e.g., sections or figure legends), or explain why this information doesn’t apply to your submission:

No grouping, randomization or masking was used in this work.

**Additional data files (“source data”)**

- We encourage you to upload relevant additional data files, such as numerical data that are represented as a graph in a figure, or as a summary table
- Where provided, these should be in the most useful format, and they can be uploaded as “Source data” files linked to a main figure or table
- Include model definition files including the full list of parameters used
- Include code used for data analysis (e.g., R, MatLab)
- Avoid stating that data files are “available upon request”

Please indicate the figures or tables for which source data files have been provided:

Kinetics analysis

We found that the apparent kinetics of the Magnets variants reported in this study fit well to an exponential decay model. We used the curve-fitting tool (cftool) in MATLAB to determine the kinetic rate constants, τON and τOFF, by fitting the curve to the following equation:

$$S\left[ t \right]=S_{0}+\Delta S e^{-\frac{t-t_{0}}{\tau}}$$

Where $S=\frac{\Delta f}{f_{\circ}}, t_{0}$is time at which the light is turned on or off (for on- or off-kinetics, respectively), S_0_ is S at time t_0_, and $\Delta S=S_{0}-S(\infty)$. During the fitting process, each point is given a weight proportional to $\frac{1}{{s.e.m.}^{2}}$. The parameters of the fit can be found in Supplementary Table 4. For all the datasets acquired in this work, the R^2^’s obtained for exponential fits are always larger than 0.86 with a median of 0.98.
